# Supplementary material for: Pulmonary fibrosis distal airway epithelia are dynamically and structurally dysfunctional
Source: Nat Commun. 2021 Jul 27;12:4566. doi: 10.1038/s41467-021-24853-8 (PMC8316442; doi:10.1038/s41467-021-24853-8)
Supplement: Supplementary file 1 — Supplementary Information [file 41467_2021_24853_MOESM1_ESM.pdf]

Supplementary Information for:

Pulmonary fibrosis distal airway epithelia are dynamically and structurally dysfunctional

Stancil et al.

Supplementary Information:

Supplementary Figures 1 – 18

Supplementary Tables 1 – 3

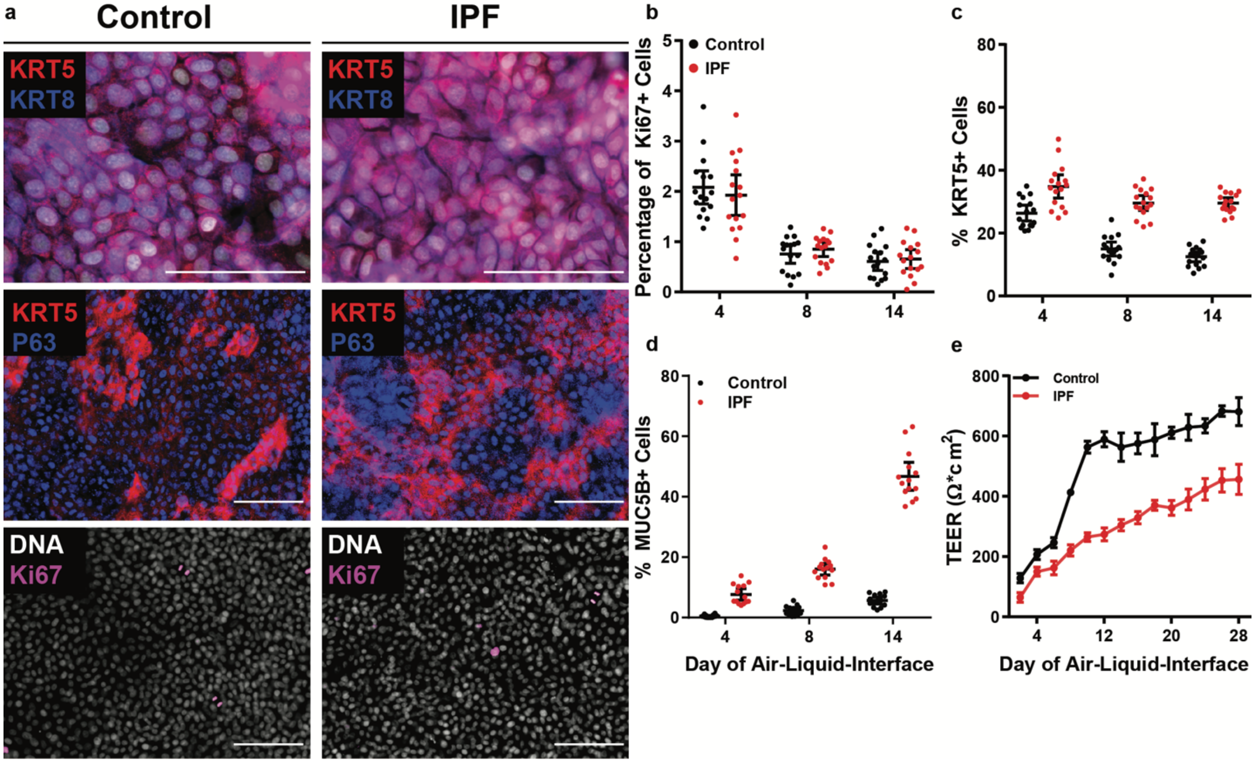

Supplementary Fig. 1. Similar basal cell populations in control and IPF with emergent differences after establishing air-liquid-interface. (A) Immunofluorescence of KRT5/KRT8, KRT5/Pp63, and Ki67 on ALI day 0. (B) Percentage of Ki67+ cells, (C) percentage of KRT5+ cells, (D) percentage of MUC5B+ cells at days 4, 8, and 14 of ALI. (E) Transepithelial electrical resistance across ALI. Shown: mean ± 95% confidence interval for n = 3 donors (control epithelia) and n = 4 donors (IPF epithelia) with ≥3 replicates and scale bars representing 100 μm.

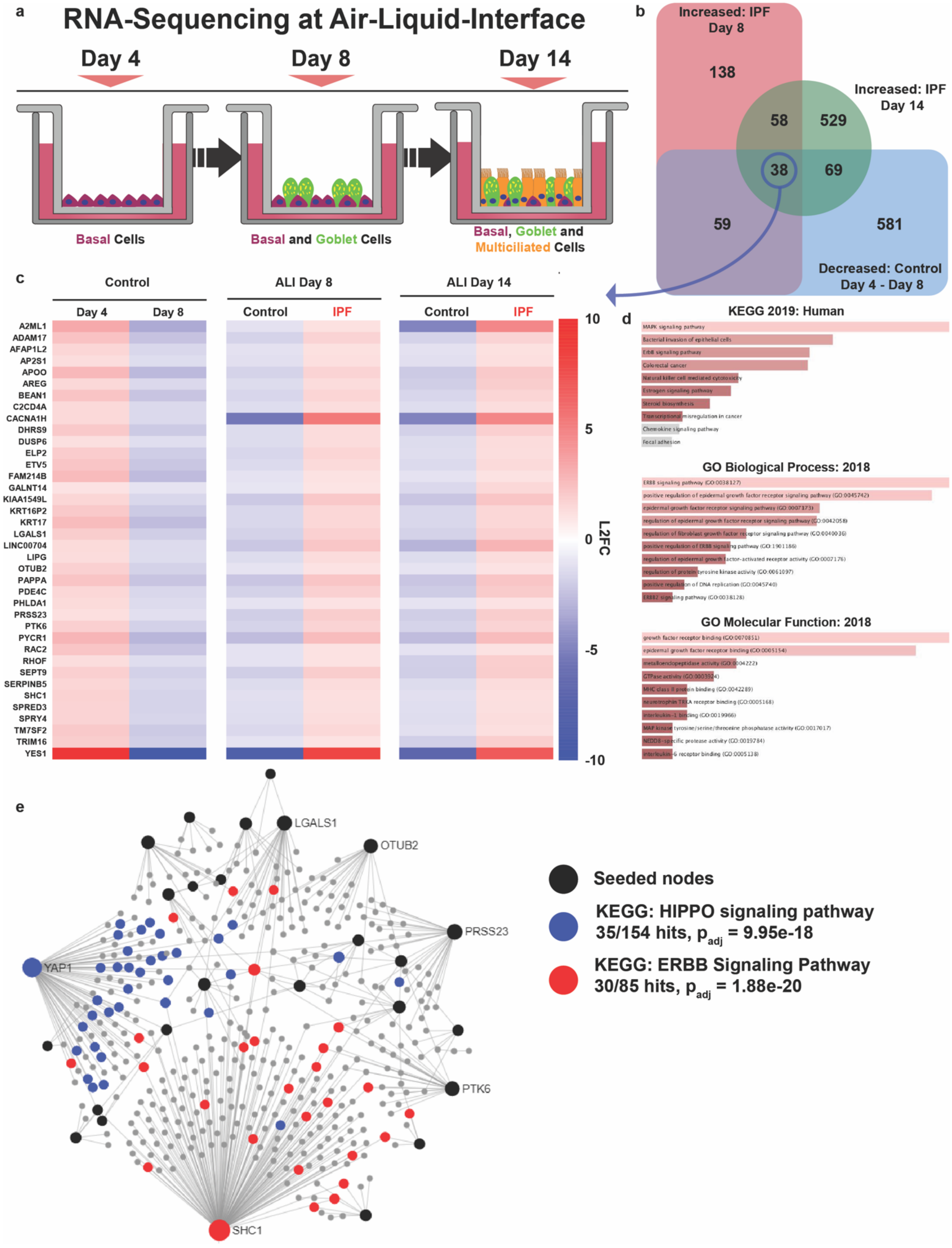
a
RNA-Sequencing at Air-Liquid-Interface
Day 4
Day 8
Day 14
Basal Cells
Basal and Goblet Cells
Basal, Goblet and Multiciliated Cells
b
Increased: IPF Day 8
Increased: IPF Day 14
Decreased: Control Day 4 - Day 8
138
58
529
38
69
59
581
c
Control
ALI Day 8
ALI Day 14
Day 4
Day 8
Control
IPF
Control
IPF
A2ML1
ADAM17
AFAP1L2
AP2S1
APOO
AREG
BEAN1
C2CD4A
CACNA1H
DHRS9
DUSP6
ELP2
ETV5
FAM214B
GALNT14
KIAA1549L
KRT16P2
KRT17
LGALS1
LINC00704
LIPG
OTUB2
PAPPA
PDE4C
PHLDA1
PRSS23
PTK6
PYCR1
RAC2
RHOF
SEPT9
SERPINB5
SHC1
SPRED3
SPRY4
TM7SF2
TRIM16
YES1
10
5
0
-5
-10
L2FC
d
KEGG 2019: Human
MAPK signaling pathway
Bacterial invasion of epithelial cells
ErbB signaling pathway
Colorectal cancer
Natural killer cell mediated cytotoxicity
Estrogen signaling pathway
Steroid biosynthesis
Transcriptional misregulation in cancer
Chemokine signaling pathway
Focal adhesion
GO Biological Process: 2018
ERBB signaling pathway (GO:0038127)
positive regulation of epidermal growth factor receptor signaling pathway (GO:0045742)
epidermal growth factor receptor signaling pathway (GO:0007173)
regulation of epidermal growth factor receptor signaling pathway (GO:0042058)
regulation of fibroblast growth factor receptor signaling pathway (GO:0040036)
positive regulation of ERBB signaling pathway (GO:1901186)
regulation of epidermal growth factor-activated receptor activity (GO:0007176)
regulation of protein tyrosine kinase activity (GO:0061097)
positive regulation of DNA replication (GO:0045740)
ERBB2 signaling pathway (GO:0038128)
GO Molecular Function: 2018
growth factor receptor binding (GO:0070851)
epidermal growth factor receptor binding (GO:0005154)
metalloendopeptidase activity (GO:0004222)
GTPase activity (GO:0003924)
MHC class II protein binding (GO:0042289)
neurotrophin TRKA receptor binding (GO:0005168)
interleukin-1 binding (GO:0019966)
MAP kinase tyrosine/serine/threonine phosphatase activity (GO:0017017)
NEDD8-specific protease activity (GO:0019784)
interleukin-6 receptor binding (GO:0005138)
e
LGALS1
OTUB2
PRSS23
YAP1
PTK6
SHC1
Seeded nodes
KEGG: HIPPO signaling pathway
35/154 hits, p_adj = 9.95e-18
KEGG: ERBB Signaling Pathway
30/85 hits, p_adj = 1.88e-20

Supplementary Fig. 2. 38 genes enriched for ERBB-YAP are persistently upregulated in distal primary epithelia in the unjammed phase. (A) Schematic of timeline used for bulk RNA-sequence analysis for control and IPF cultures. (B) Venn diagram of genes downregulated from day 4 → 8 in control cells and upregulated at days 8 and 14 in IPF cells. (C) Heatmap of the 38 genes downregulated in control cells and persistently upregulated in IPF. (D) KEGG and Gene ontology (biological process and molecular function) for the 38 genes associated with the unjammed state. (E) Network analysis of 38 seeded genes, black nodes are seeded, blue nodes are associated with HIPPO signaling, and red nodes are associated with ERBB signaling.

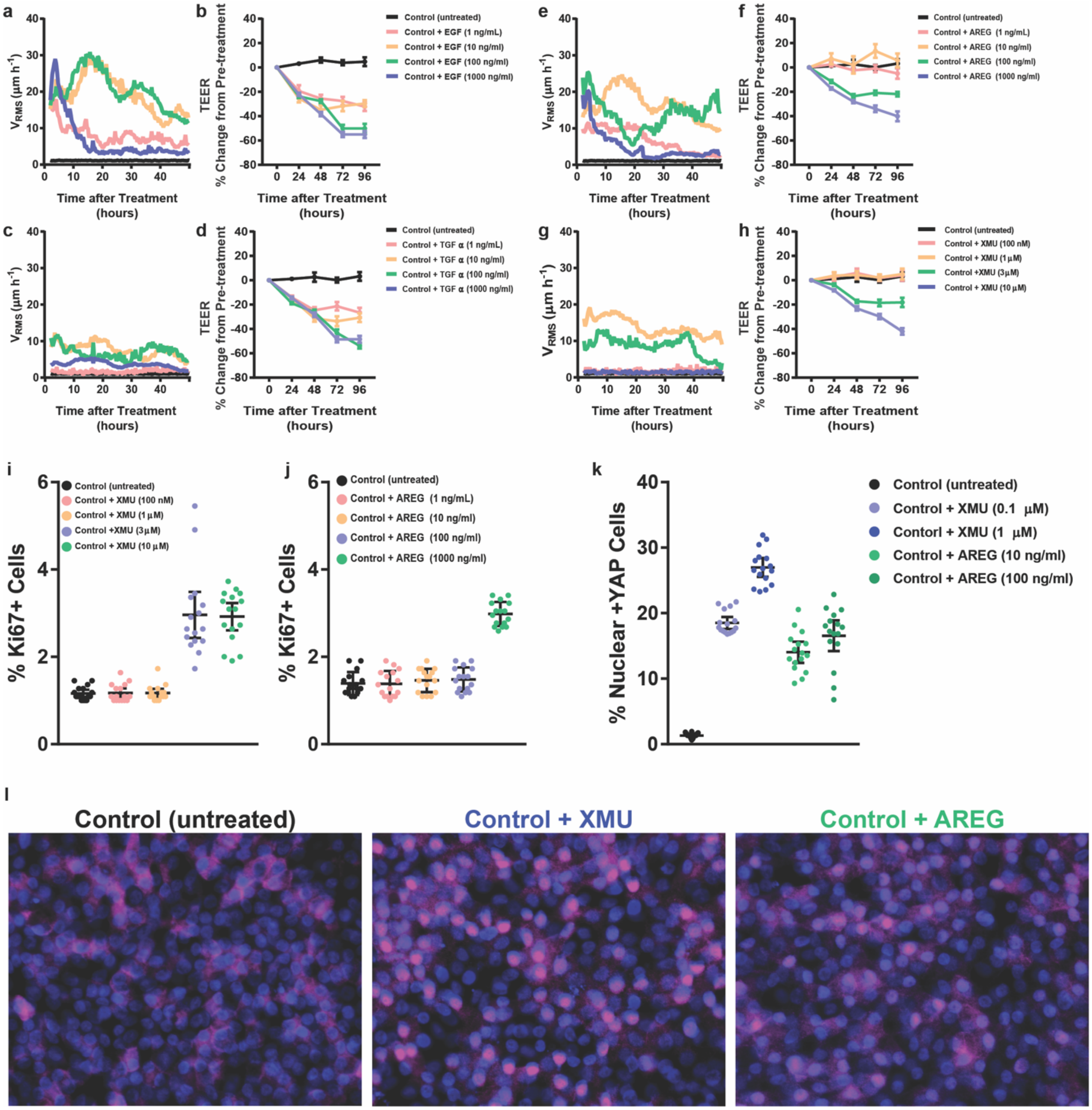

Supplementary Fig. 3. AREG and YAP are sufficient to drive unjamming, but not EGF or TGFa. (A) Root mean squared velocity (V_{RMS}), (B) TEER of control distal epithelia treated EGF. (C) V_{RMS}, (D) TEER of control distal epithelia treated with TGFα. (E) V_{RMS}, (F) TEER of control distal epithelia treated with AREG. (G) V_{RMS}, (H) TEER of control distal epithelia treated with XMU-MP-1. (I) Percentage of Ki67+ distal epithelia 48 hours after treatment with XMU-MP-1, or (J) 48 hours after treatment with AREG. (K) Percentage of YAP nuclear positive epithelia 48 hours after treatment with XMU-MP-1 or AREG. (L) Representative images of YAP nuclear localization 48 hours after XMU-MP-1 or AREG. Shown: mean ± 95% confidence interval for n = 3 donors

(control epithelia) and n = 4 (IPF epithelia) with ≥3 replicates and scale bars representing 100 μm.

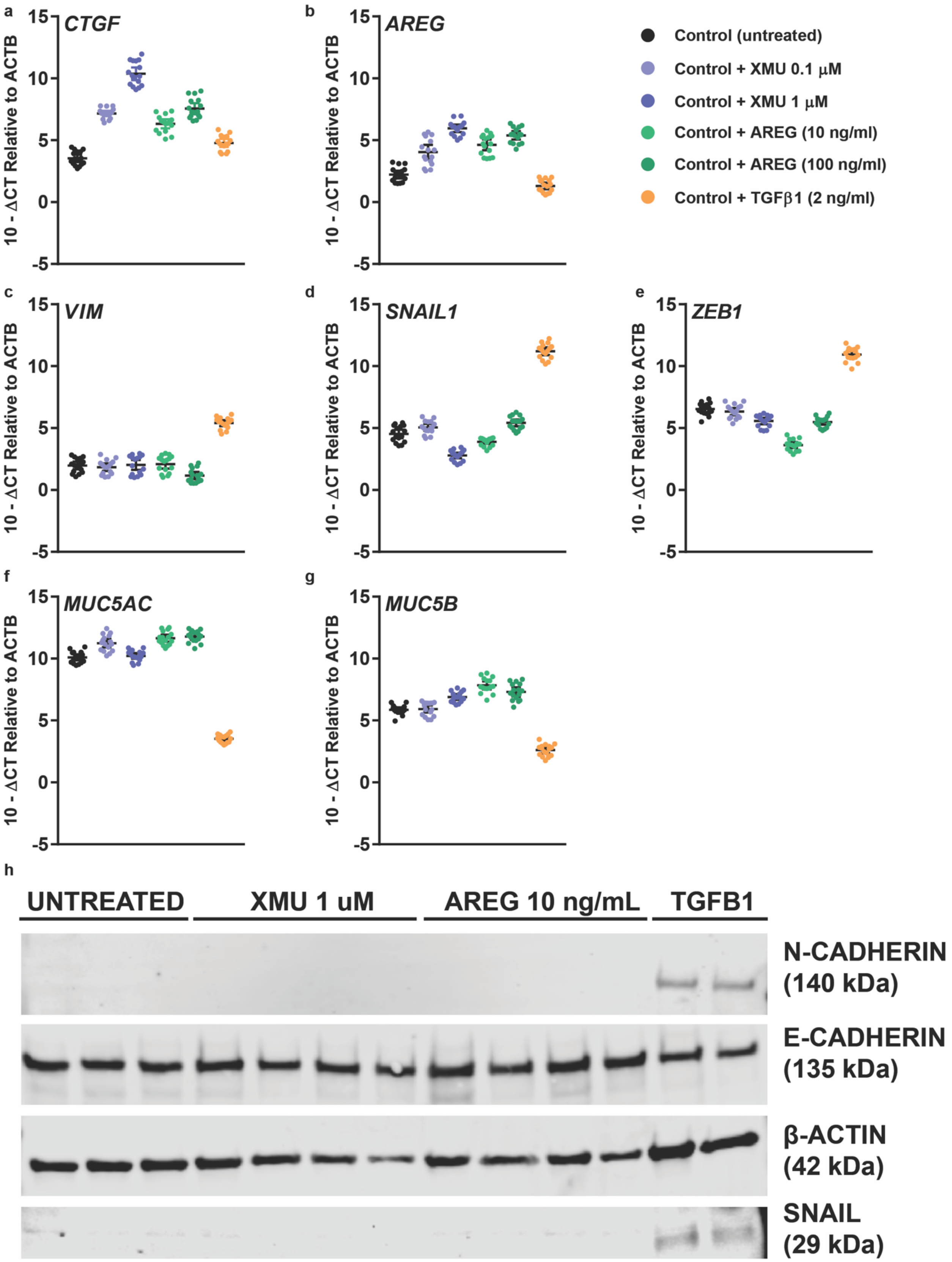

a
CTGF
b
AREG
Control (untreated)
Control + XMU 0.1 μM
Control + XMU 1 μM
Control + AREG (10 ng/ml)
Control + AREG (100 ng/ml)
Control + TGFβ1 (2 ng/ml)
c
VIM
d
SNAIL1
e
ZEB1
f
MUC5AC
g
MUC5B
10 - ΔCT Relative to ACTB
15
10
5
0
-5
h
UNTREATED
XMU 1 uM
AREG 10 ng/mL
TGFB1
N-CADHERIN (140 kDa)
E-CADHERIN (135 kDa)
β-ACTIN (42 kDa)
SNAIL (29 kDa)

41

Supplementary Fig. 4. EGFR-YAP induced unjamming does not induce a partial epithelial-to-mesenchymal transition. (**A-G**) Gene expression of YAP target genes (*CTGF, AREG*), EMT-associated genes (VIM, SNAIL1, ZEB1), and airway mucin genes (*MUC5AC, MUC5B*) 96 hours after chronic treatment with XMU (0.1 or 1 μM) AREG (10 or 100 ng/ml) or TGFβ1 (2 ng/ml), error bars represent 95% CI. (**H**) Western blot for N-cadherin, E-cadherin, β-actin, or Snail in control distal epithelia 96 hours after chronic treatment with XMU (0.1 or 1 μM) AREG (10 or 100 ng/ml) or TGFβ1 (2 ng/ml). Shown: mean $\pm$ 95% confidence interval for n = 3 donors (control epithelia) and n = 4 donors (IPF epithelia) with $\geq$2 replicates. Uncropped versions, ladder visible version of the blot is available in the accompanying online Source Data file. The loading control was ran on a separate gel from the other blotted proteins.

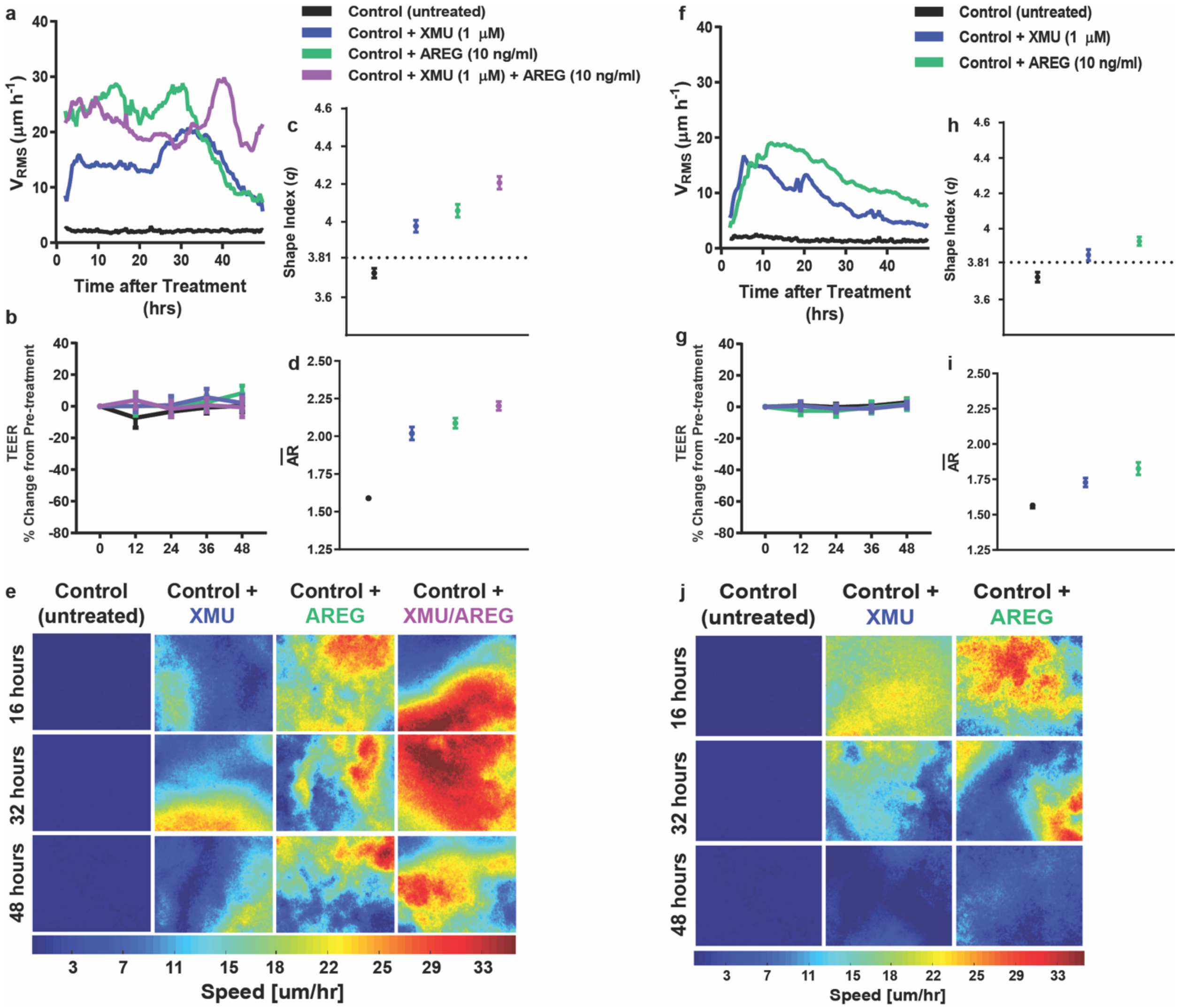

Supplementary Fig. 5. Combined AREG+YAP treatment potentiates the unjammed phase and EGFR-YAP driven unjamming occurs regardless of monolayer maturity. (**A**) Root mean squared velocity (V_{RMS}) and (**B**) Transepithelial electrical resistance (TEER) of control distal epithelia treated with XMU, AREG or XMU and AREG, error bars represent standard error of the mean (SEM). (**C**) Shape index and (**D**) aspect ratio (AR) for control distal epithelia 48 hours after treatment with XMU, AREG or XMU and AREG, error bars represent 95% CI. (**E**) Representative heatmap of control distal epithelia every 16 hours after treatment. (**F**) V_{RMS} and (**G**) TEER of control distal epithelia at day 28 treated with XMU or AREG, error bars represent SEM. (**H**) Shape index and (**I**) AR of control distal epithelia at day 28 treated with XMU or AREG, error bars represent 95% CI. (**J**) Representative heatmap of control distal epithelia on day 28 every 16 hours after treatment. Shown: mean $\pm$ 95% confidence interval for n = 4 donors (control epithelia) with $\geq$3 replicates.

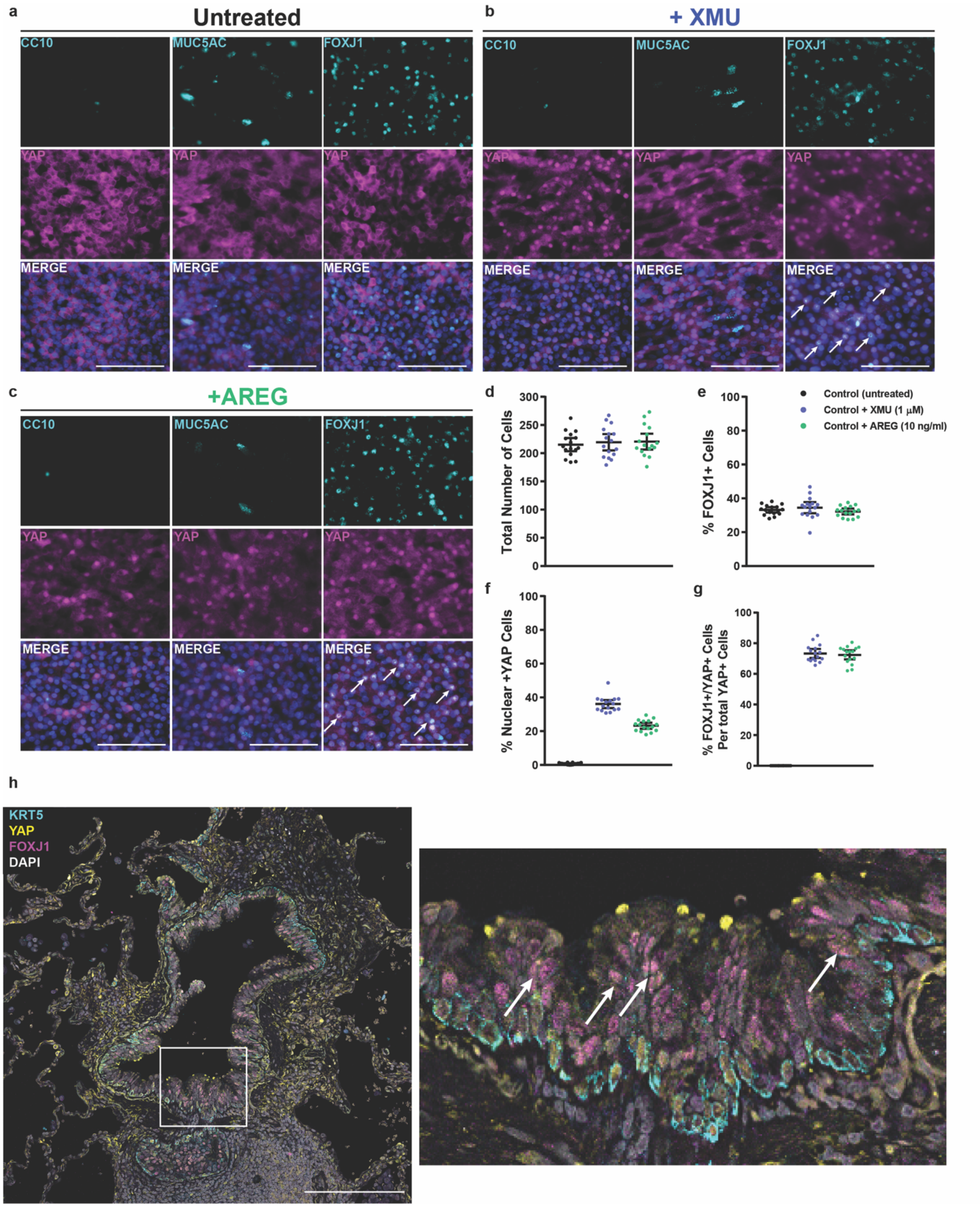

Supplementary Fig. 6. EGFR-YAP activation leads to YAP nuclear localization in FOXJ1+ cells in vitro and are present in IPF in areas of minimal fibrosis in vivo*.* (**A-C**) Control distal epithelia untreated or treated with XMU or AREG, stained for YAP and club (CC10), goblet

(MUC5AC), or ciliated (FOXJ1) cell markers and white arrows indicating examples of co-localization of FOXJ1 and YAP. (**D**) Total number of, (**E**) percentage of FOXJ1 positive cells, (**F**) percentage of YAP positive cells, and (**G**) percentage of FOXJ1/YAP co-positive cells per field of view, error bars represent 95% CI. (**H**) Human lung sections from an IPF patient stained for KRT5, FOXJ1, and YAP. Shown: mean $\pm$ 95% confidence interval for n = 4 donors (control epithelia) with $\geq$3 replicates and scale bars representing 100 μm.

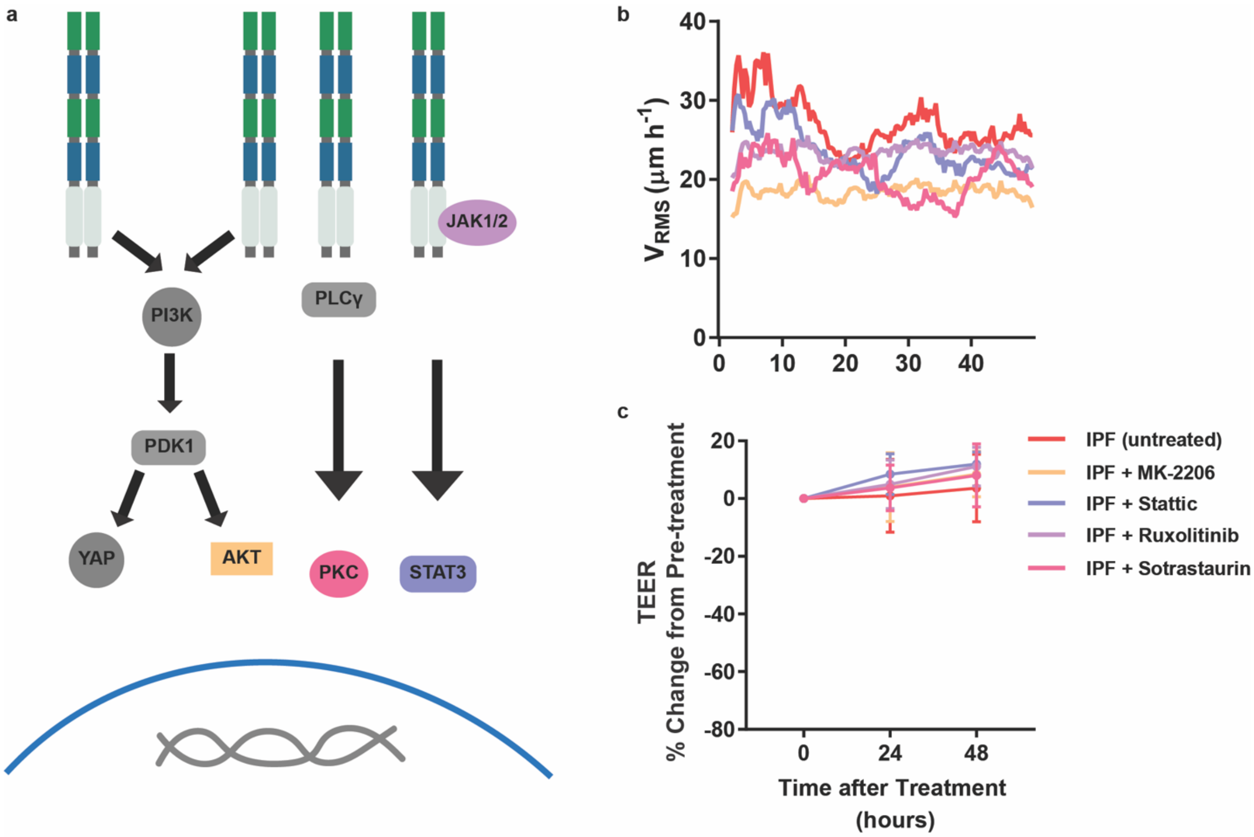

Supplementary Fig. 7. In IPF epithelia, inhibition of AKT, JAK, STAT, or PKC does not induce jamming. (**A**) Schematic of downstream ERBB-YAP signaling. (**B**) Root mean squared velocity and (**C**) Transepithelial electrical resistance of IPF distal epithelia treated with MK2206, Stattic, Ruxolitinib, or Sotrastaurin, error bars represent standard error of the mean. Shown: mean ± 95% confidence interval for n = 4 donors (IPF epithelia) with ≥3 replicates.

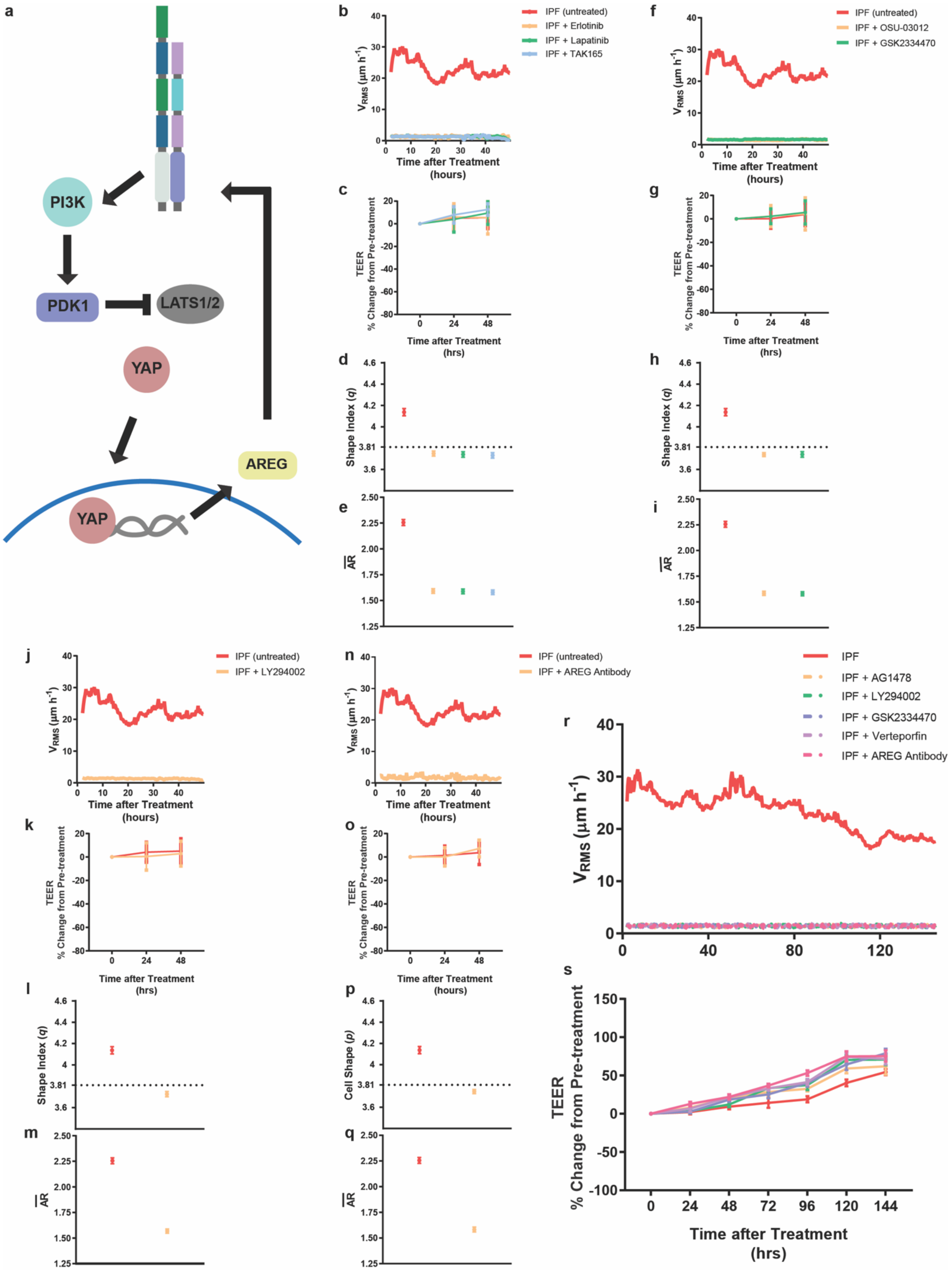
a
PI3K
PDK1
LATS1/2
YAP
AREG
YAP
b
IPF (untreated)
IPF + Erlotinib
IPF + Lapatinib
IPF + TAK165
V_{RMS} (μm h^{-1})
Time after Treatment (hours)
c
TEER % Change from Pre-treatment
Time after Treatment (hrs)
d
Shape Index (q)
3.81
e
$\overline{AR}$
f
IPF (untreated)
IPF + OSU-03012
IPF + GSK2334470
V_{RMS} (μm h^{-1})
Time after Treatment (hours)
g
TEER % Change from Pre-treatment
Time after Treatment (hrs)
h
Shape Index (q)
3.81
i
$\overline{AR}$
j
IPF (untreated)
IPF + LY294002
V_{RMS} (μm h^{-1})
Time after Treatment (hours)
k
TEER % Change from Pre-treatment
Time after Treatment (hrs)
l
Shape Index (q)
3.81
m
$\overline{AR}$
n
IPF (untreated)
IPF + AREG Antibody
V_{RMS} (μm h^{-1})
Time after Treatment (hours)
o
TEER % Change from Pre-treatment
Time after Treatment (hours)
p
Cell Shape (p)
3.81
q
$\overline{AR}$
IPF
IPF + AG1478
IPF + LY294002
IPF + GSK2334470
IPF + Verteporfin
IPF + AREG Antibody
r
V_{RMS} (μm h^{-1})
s
TEER % Change from Pre-treatment
Time after Treatment (hrs)

Supplementary Fig. 8. Inhibition of the ERBB-YAP axis induces jamming in IPF distal epithelia. (**A**) Schematic of the ERBB-YAP signaling cascade. (**B**) Root mean square velocity (V_{RMS}), (**C**) Transepithelial electrical resistance (TEER), (**D**) shape index, and (**E**) aspect ratio (AR) of IPF distal epithelia 48 hours after treatment with Erlotinib, Lapatinib, Mubritinib (EGFR/ERBB inhibitors). (**F**) V_{RMS}, (**G**) TEER, (**H**) shape index, and (**I**) AR of IPF distal epithelia 48 hours after treatment with OSU03012 or GSK2334470 (PDK1 inhibitors). (**J**) V_{RMS}, (**K**) TEER, (**L**) shape index*,* and (**M**) AR of IPF distal epithelia 48 hours after treatment with LY294002 (PI3K inhibitor). (**N**) V_{RMS}, (**O**) TEER, (**P**) shape index and, (**Q**) AR of IPF distal epithelia 48 hours after treatment with Amphiregulin neutralizing antibody. (**R**) V_{RMS} and (**S**) TEER of IPF distal epithelia followed for 144 hours after a single treatment with AG1478, LY294002, GSK2334470, Verteporfin, or Amphiregulin neutralizing antibody. Shown: mean $\pm$ 95% confidence interval for n = 4 donors (IPF epithelia) with $\geq$3 replicates.

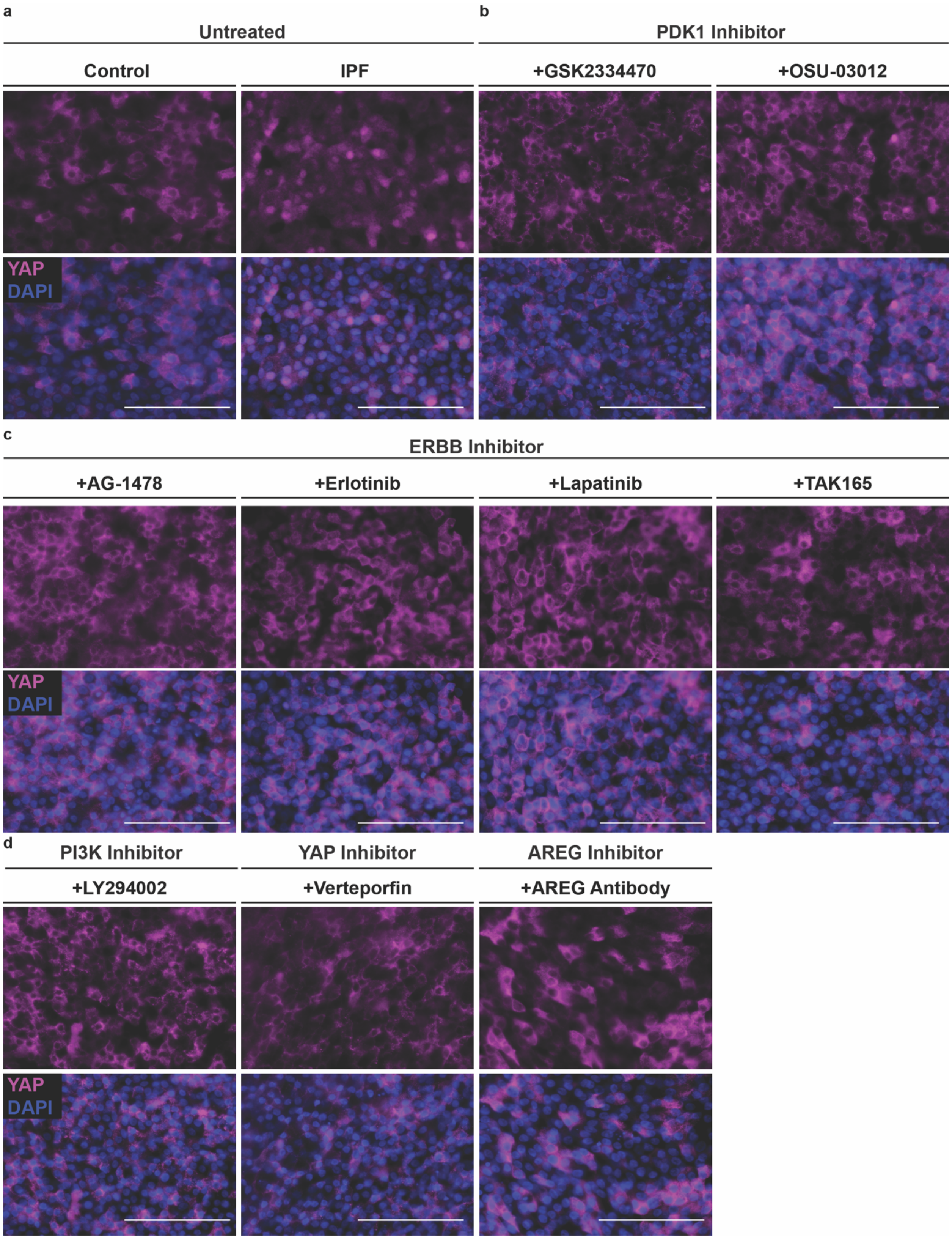

Supplementary Fig. 9. Inhibition of the ERBB-YAP axis in IPF distal epithelia results in YAP nuclear exclusion. (**A**) Control and IPF untreated distal epithelial cultures, (**B**) GSK2334470,

OSU03012, (**C**) AG1478, Erlotinib, Lapatinib, Mubritinib, (**D**) LY294002, Verteporfin, or AREG neutralizing antibody immunofluorescence for YAP 48 hours after treatment of IPF distal epithelia, scale bars represent 100 μm. Images display representative images from n = 3 donors with 2 replicates.

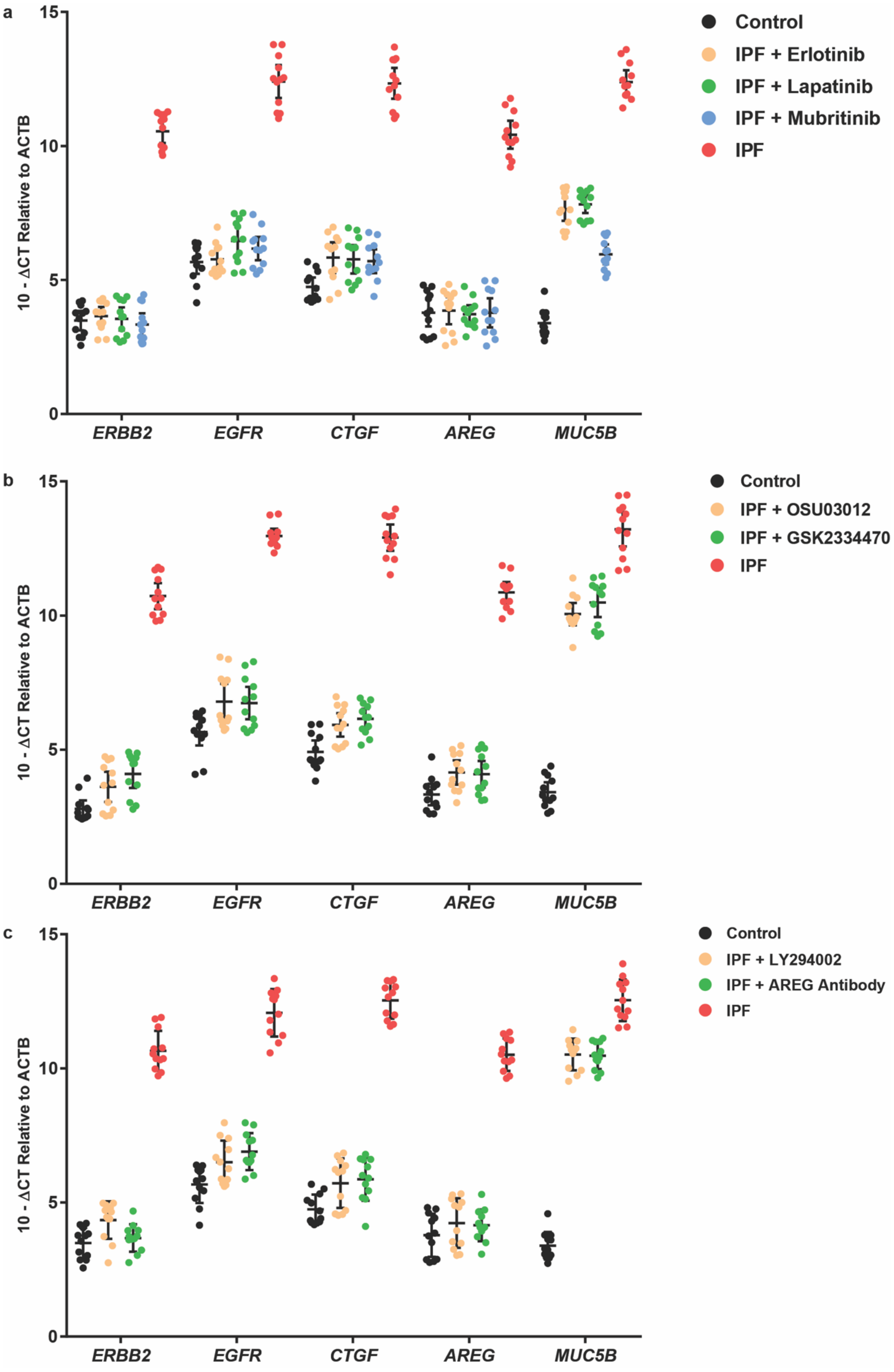
a
b
c
10 - ΔCT Relative to ACTB
0
5
10
15
ERBB2
EGFR
CTGF
AREG
MUC5B
Control
IPF + Erlotinib
IPF + Lapatinib
IPF + Mubritinib
IPF
IPF + OSU03012
IPF + GSK2334470
IPF + LY294002
IPF + AREG Antibody

Supplementary Fig. 10. Inhibition of the ERBB-YAP axis in IPF distal epithelia decreases ERBB-YAP target gene expression. Gene expression of ERBB receptors (*EGFR, ERBB2*), YAP target genes (*CTGF, AREG*), and *MUC5B* 48 hours after treatment with (**A**) Erlotinib, Lapatinib, or Mubritinib, (**B**) GSK2334470 or OSU03012, or (**C**) LY294002, Verteporfin, or AREG neutralizing antibody. Shown: mean $\pm$ 95% confidence interval for n = 3 donors (control epithelia) and n = 4 (IPF epithelia) with $\geq$3 replicates.

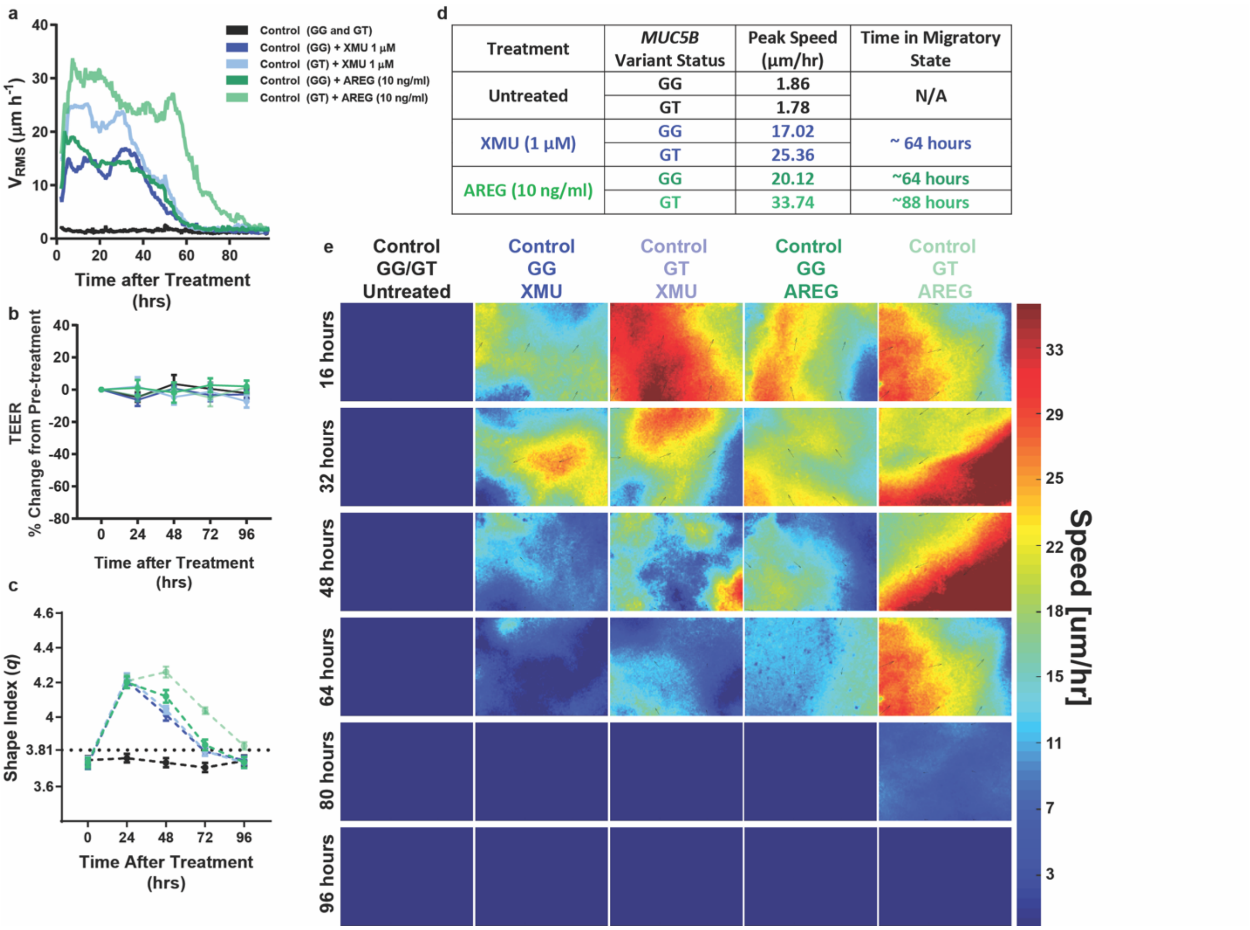

Supplementary Fig. 11. rs35705950 potentiates EGFR-driven unjamming in healthy distal epithelia. (**A**) Root mean squared velocity, (**B**) transepithelial electrical resistance, and (**C**) shape index of control distal epithelia segregated by their rs35705950 promoter variant status for 96 hours after treatment, error bars represent 95% confidence intervals. (**D**) Table of peak speed and length of time in a migratory state for control distal epithelia after treatment segregated by their rs35705950 promoter variant status. (**E**) Representative heatmaps of control distal epithelia every 16 hours after treatment. Shown: mean ± 95% confidence interval for n = 4 donors (GG control epithelia) and n = 4 donors (GT control epithelia) with ≥3 replicates.

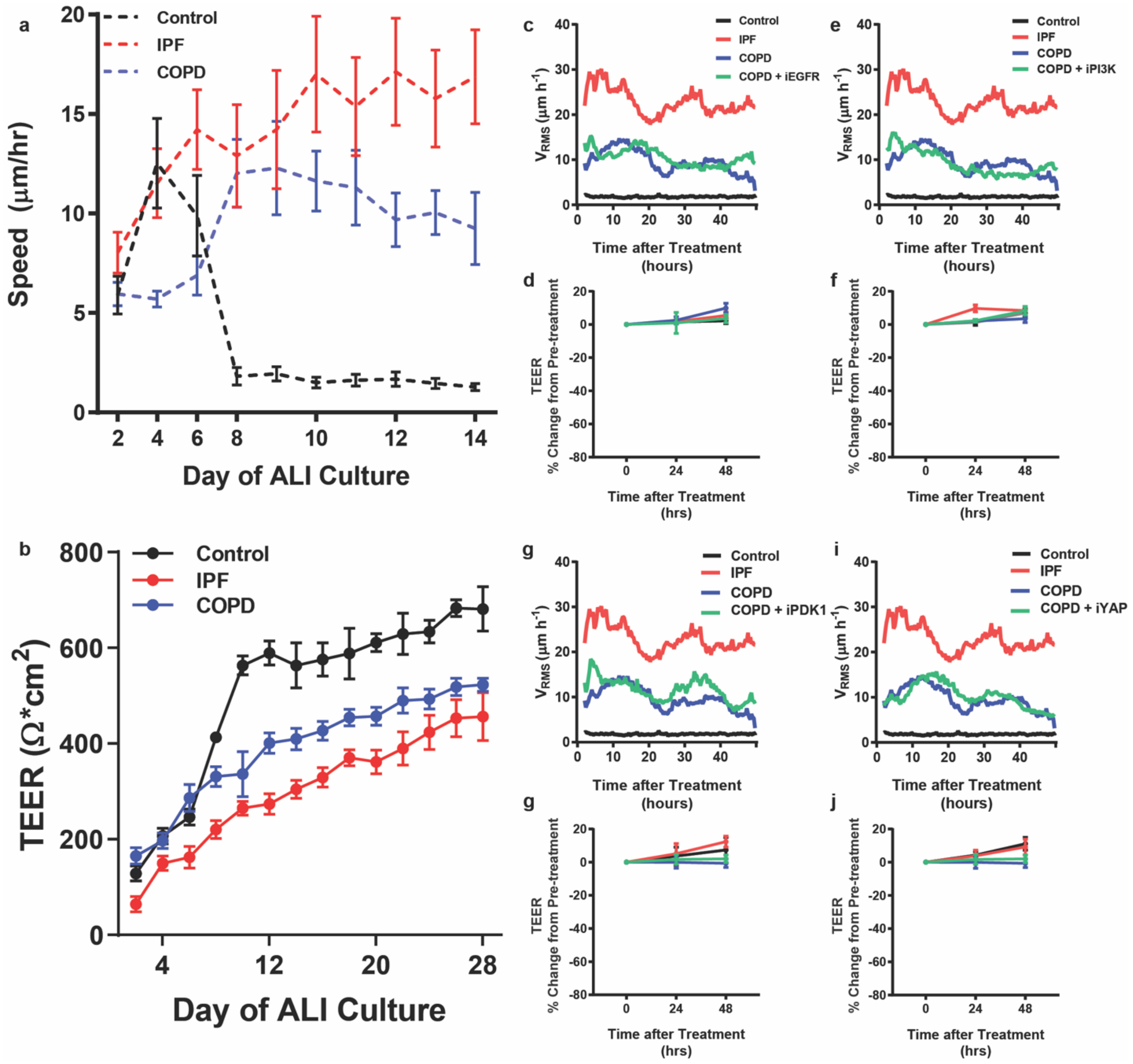

Supplementary Fig. 12. The unjammed phase in COPD is not responsive to ERBB-YAP inhibition. (**A**) Average speed (μm/hr) and (**B**) transepithelial electrical resistance (TEER) of control, IPF and COPD distal epithelia. (**C**) Root mean square velocity (V_{RMS}) and (**D**) TEER of COPD distal epithelia after AG1478 treatment. (**E**) V_{RMS} and (**F**) TEER of COPD distal epithelia after LY294002 treatment. (**G**) V_{RMS} and (**H**) TEER of COPD distal epithelia after OSU03012 treatment. (**I**) V_{RMS} and (**J**) TEER of COPD distal epithelia after Verteporfin treatment. Shown: mean ± 95% confidence interval for n = 3 (control epithelia), n = 4 (IPF epithelia), and n = 3 (COPD epithelia) donors with ≥3 replicates.

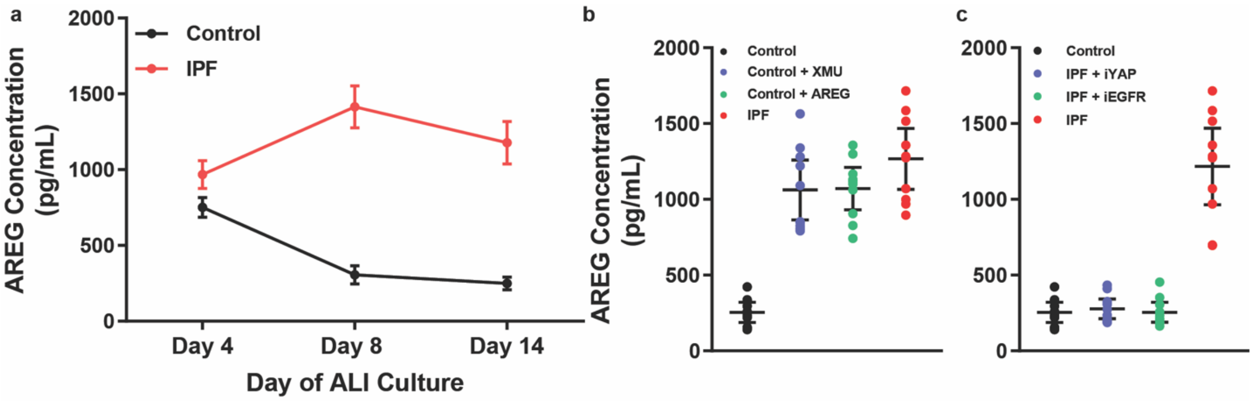

Supplementary Fig. 13. AREG secretion is modulated by altering the physical state of the epithelia. (**A**) Control and IPF distal epithelial AREG concentration throughout ALI. (**B**) AREG concentration after control distal epithelial induced unjamming. (**C**) AREG concentration after IPF distal epithelial induced jamming. Shown: mean ± 95% confidence interval for n = 3 (control epithelia) and n = 4 (IPF epithelia) donors with ≥3 replicates.

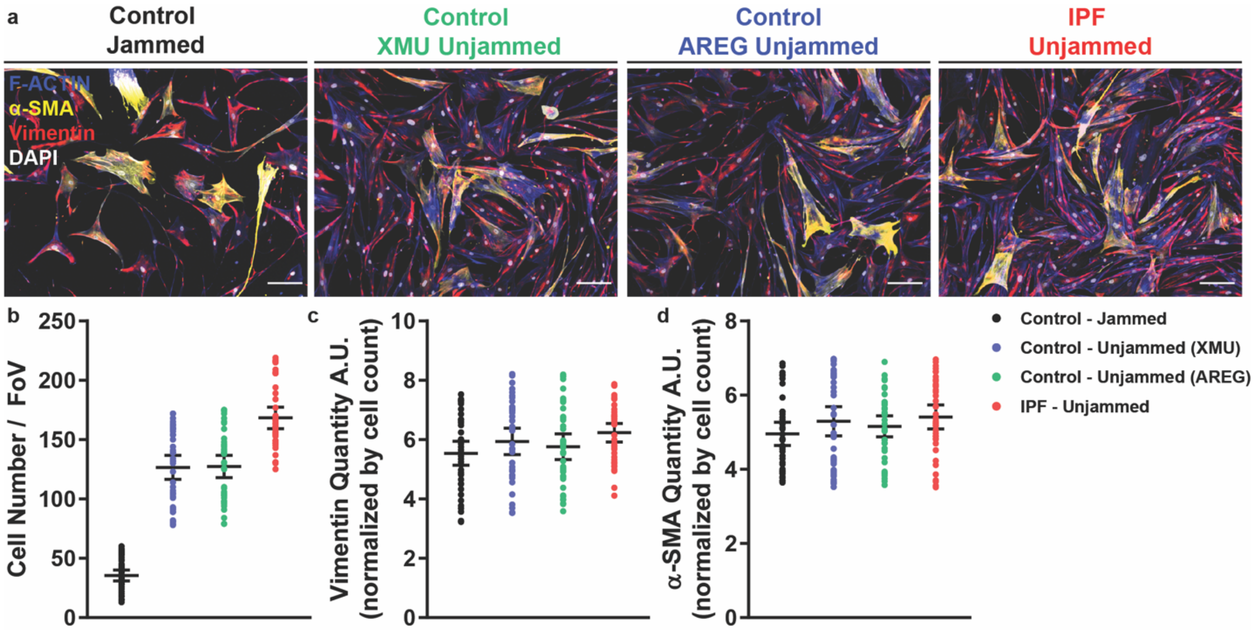

Supplementary Fig. 14. Human lung fibroblasts on plastic fail to demonstrate robust treatment differences. (**A**) Immunofluorescence of primary human lung fibroblast (HLF) seeded on tissue culture plastic 72 hours after treatment with control-jammed, control-YAP unjammed, control-AREG unjammed, or IPF-unjammed distal epithelial media, scale bar represents 100 μm. (**B**) Cell number, (**C**) vimentin, and (**D**) alpha-SMA mean fluorescence of treated HLF. Shown: mean ± 95% confidence interval for n = 4 donors with ≥2 replicates per donor.

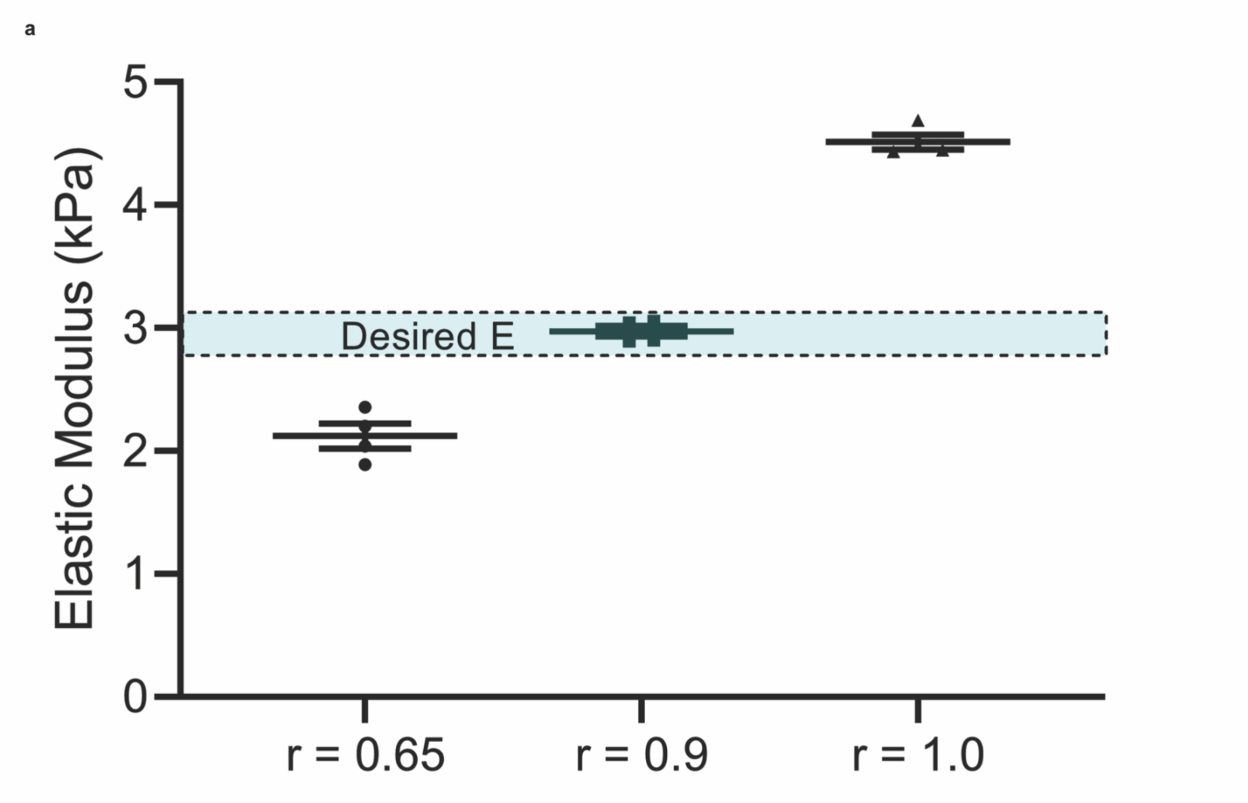

Supplementary Fig. 15. Synthesized hydrogel stiffness is 3kPa. (**A**) Elastic modulus of synthesized hydrogel, r represents the ratio of macromer end groups that are crosslinked. A hydrogel with a r of 0.9 has 90% of end groups crosslinked, which allows for the remaining, un-crosslinked groups, to be conjugated with adhesive peptides. Shown: Shown: mean $\pm$ 95% confidence interval for n = 4

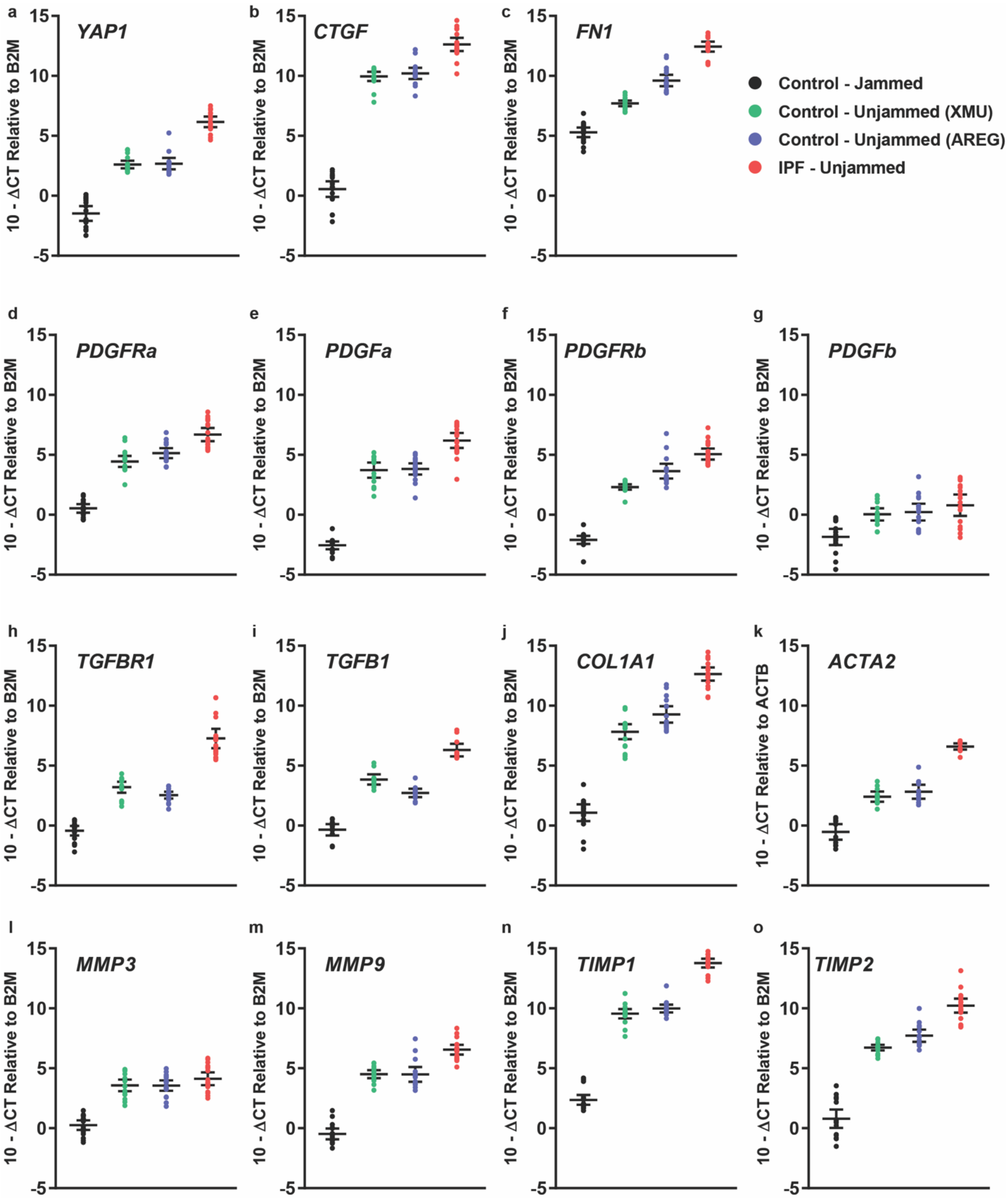

Supplementary Fig. 16. The unjammed phase induces pro-fibrotic gene expression in primary human lung fibroblasts. (**A-O**) Gene expression 72 hours after treatment with distal epithelial media. Shown: mean ± 95% confidence interval for n = 4 donors with ≥2 replicates per donor.

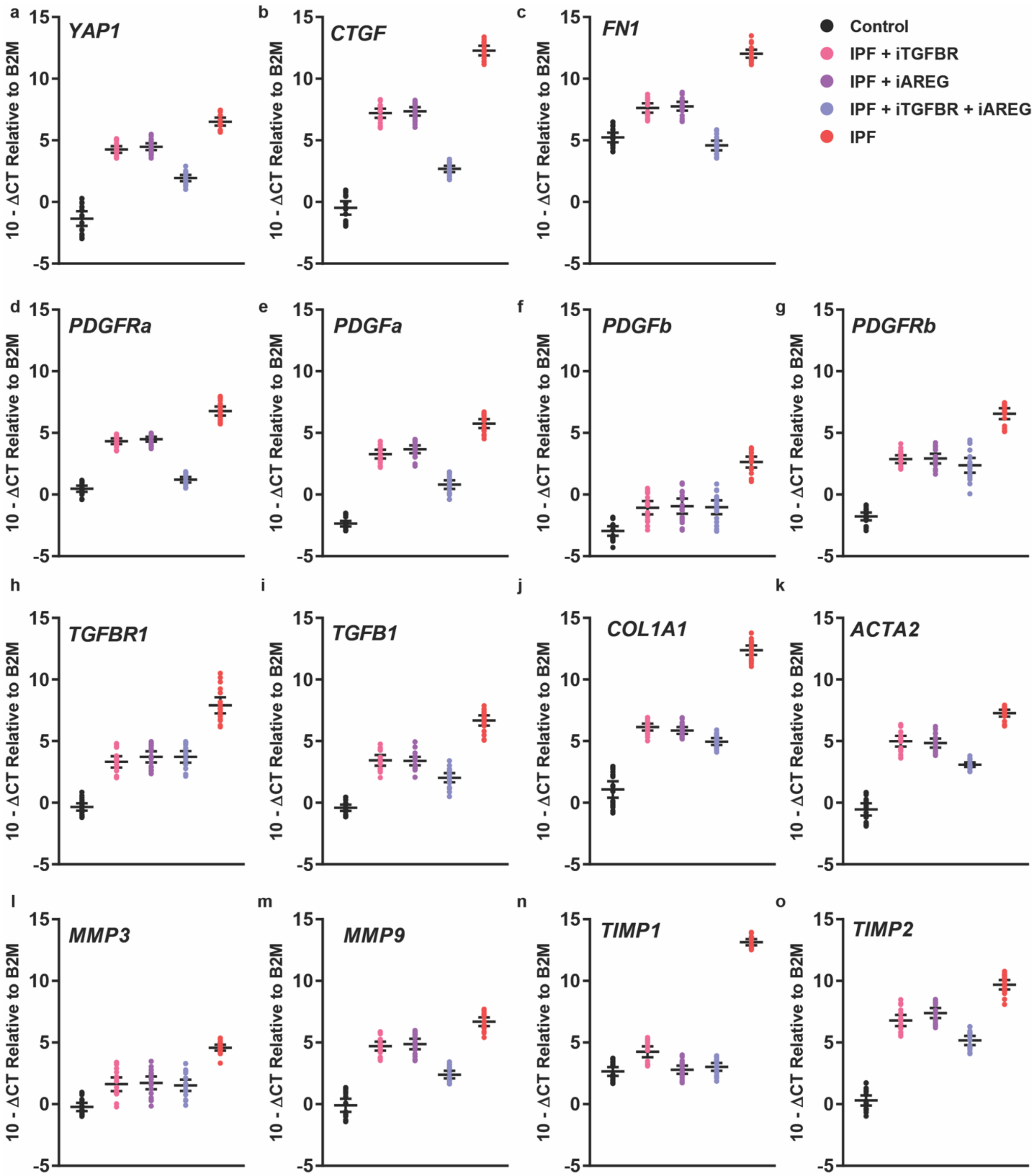

Supplementary Fig. 17. Direct inhibition of human lung fibroblast attenuates unjammed induced pro-fibrotic gene expression. (**A-O**) Gene expression 72 hours after treatment with distal epithelial media. Shown: mean ± 95% confidence interval for n = 4 donors with ≥2 replicates per donor.

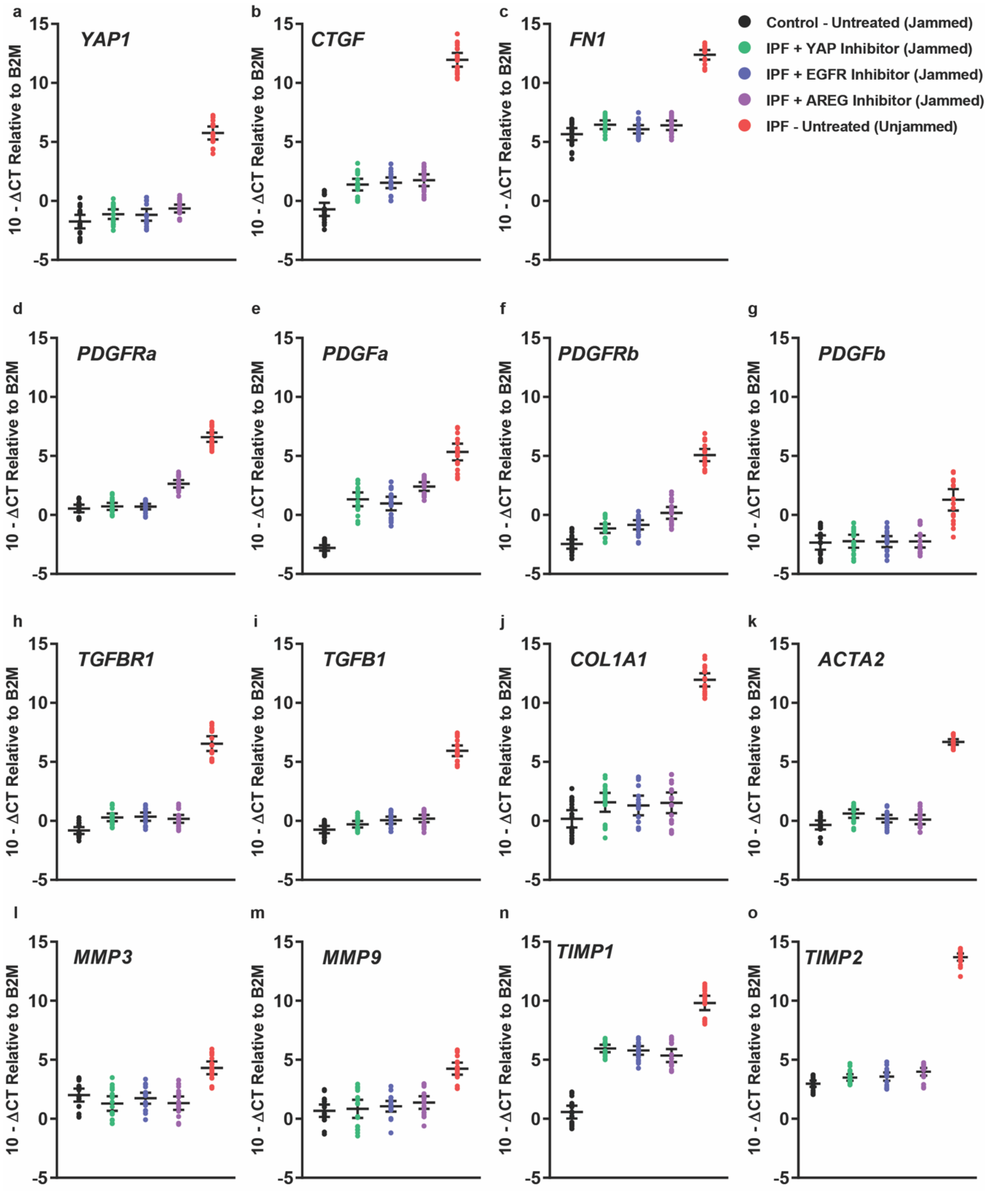

Supplementary Fig. 18. Induced jamming in IPF epithelia media rescues pro-fibrotic gene expression in human lung fibroblasts. (**A-O**) Gene expression 72 hours after treatment with distal epithelial media. Shown: mean ± 95% confidence interval for n = 4 donors with ≥2 replicates per donor.

Disease Status	Cell	Passage	Age	Sex	Smoking	Isolation Method	Region	*MUC5B* Status	Experiments
Control	Epithelial	2	62 ± 6.23	Male (7): Female (4)	Never (5): Ever (6)	Brushing	Distal Airway	GG (7): GT (4)	Jamming and RNA-Seq
Control	Fibroblast	3	66.3 ± 6.5	Male (2): Female (2)	Never (2): Ever (2)	Dissection	Distal Lung	GG (4)	Hydrogel Treatments
IPF	Epithelial	2	65 ± 5.26	Male (3): Female (1)	Never (2): Ever (2)	Brushing	Distal Airway	GG (2): GT (2)	Jamming and RNA-Seq
				Male (3)	Never (2): Ever (1)	Dissection	Distal & Proximal Airways	GG (2): GT (1)	Jamming
COPD	Epithelial	2	64.3 ± 3.7	Male (2): Female (1)	Ever (3)	Dissection	Distal Airway	GG (3)	Jamming

Supplementary Table 1. Primary cell donor information. List of cell type, passage, age, sex, smoking history, isolation method, region and *MUC5B* variant status used for all experiments.

Common "Jamming" Related Genes Control Day 4 → Day 8 of ALI		
Gene Name	Control Day 4 (L2FC)	Control Day 8 (L2FC)
A2ML1	3.35968	-3.35968
ADAM17	2.36923	-2.36923
AFAP1L2	1.57346	-1.57346
AP2S1	1.32405	-1.32405
APOO	2.73311	-2.73311
AREG	1.51958	-1.51958
BEAN1	2.29793	-2.29793
C2CD4A	1.45859	-1.45859
CACNA1H	1.48804	-1.48804
DHRS9	2.10621	-2.10621
DUSP6	1.24182	-1.24182
ELP2	1.98565	-1.98565
ETV5	2.21254	-2.21254
FAM214B	2.66521	-2.66521
GALNT14	1.02217	-1.02217
KIAA1549L	1.59375	-1.59375
KRT16P2	2.11183	-2.11183
KRT17	2.59168	-2.59168
LGALS1	1.94015	-1.94015
LINC00704	1.39032	-1.39032
LIPG	1.3435	-1.3435
OTUB2	1.79158	-1.79158
PAPPA	2.30254	-2.30254
PDE4C	1.56141	-1.56141
PHLDA1	1.38825	-1.38825
PRSS23	1.19527	-1.19527
PTK6	1.81366	-1.81366
PYCR1	2.84926	-2.84926
RAC2	2.26525	-2.26525
RHOF	1.25856	-1.25856
SEPT9	1.80608	-1.80608
SERPINB5	1.60017	-1.60017
SCH1	1.72804	-1.72804
SPRED3	1.69745	-1.69745
SPRY4	1.65284	-1.65284
TM7SF2	2.10907	-2.10907
TRIM16	1.9556	-1.9556
YES1	9.6906	-9.6906

Common "Jamming" Related Genes Day 8 of ALI		
Gene Name	Control Day 8 (L2FC)	IPF Day 8 (L2FC)
A2ML1	-1.130477	1.130477
ADAM17	-1.701	1.701
AFAP1L2	-1.11	1.11
AP2S1	-1.206	1.206
APOO	-1.695038	1.695038
AREG	-1.337	1.337
BEAN1	-1.634	1.634
C2CD4A	-1.305	1.305
CACNA1H	-5.393318	5.393318
DHRS9	-1.359514	1.359514
DUSP6	-1.341	1.341
ELP2	-1.620609	1.620609
ETV5	-1.417	1.417
FAM214B	-1.066	1.066
GALNT14	-1.006269	1.006269
KIAA1549L	-2.124072	2.124072
KRT16P2	-1.567	1.567
KRT17	-1.531	1.531
LGALS1	-1.785	1.785
LINC00704	-2.176459	2.176459
LIPG	-1.367	1.367
OTUB2	-1.031	1.031
PAPPA	-2.261	2.261
PDE4C	-1.78453	1.78453
PHLDA1	-1.035	1.035
PRSS23	-1.96	1.96
PTK6	-1.717	1.717
PYCR1	-2.591	2.591
RAC2	-1.096	1.096
RHOF	-1.093947	1.093947
SEPT9	-1.931	1.931
SERPINB5	-1.371	1.371
SCH1	-1.185	1.185
SPRED3	-1.254	1.254
SPRY4	-1.289	1.289
TM7SF2	-1.493291	1.493291
TRIM16	-1.088076	1.088076
YES1	-8.143988	8.143988

Common "Jamming" Related Genes Day 14 of ALI		
Gene Name	Control Day 14 (L2FC)	IPF Day 14 (L2FC)
A2ML1	-4.822781	4.822781
ADAM17	-1.701	1.701
AFAP1L2	-1.11	1.11
AP2S1	-1.206	1.206
APOO	-2.093187	2.093187
AREG	-1.9431	1.9431
BEAN1	-1.634	1.634
C2CD4A	-1.305	1.305
CACNA1H	-4.893584	4.893584
DHRS9	-1.83051	1.83051
DUSP6	-1.341	1.341
ELP2	-1.272051	1.272051
ETV5	-1.417	1.417
FAM214B	-1.066	1.066
GALNT14	-1.502481	1.502481
KIAA1549L	-2.25629	2.25629
KRT16P2	-1.567	1.567
KRT17	-1.531	1.531
LGALS1	-1.785	1.785
LINC00704	-2.916479	2.916479
LIPG	-1.367	1.367
OTUB2	-1.031	1.031
PAPPA	-2.261	2.261
PDE4C	-2.027821	2.027821
PHLDA1	-1.035	1.035
PRSS23	-1.96	1.96
PTK6	-1.717	1.717
PYCR1	-2.591	2.591
RAC2	-1.096	1.096
RHOF	-1.883034	1.883034
SEPT9	-1.931	1.931
SERPINB5	-1.371	1.371
SCH1	-1.185	1.185
SPRED3	-1.254	1.254
SPRY4	-1.289	1.289
TM7SF2	-1.189712	1.189712
TRIM16	-1.900963	1.900963
YES1	-6.948261	6.948261

Supplementary Table 2. Genes correlated with the jamming transition in primary human bronchiolar epithelial cells. Genes down regulated from day 4 → 8 in control distal epithelia that are upregulated in IPF distal epithelia at days 8 and 14 of ALI.

Gene	Product ID
ACTB	Hs01060665_g1
MUC5B	Hs00861595_m1
MUC5AC	Hs01365616_m1
EGFR	Hs0176090_m1
ERBB2	Hs01001580_m1
ZEB1	Hs00232783_m1
SNAI1	Hs00195591_m1
VIM	Hs00185584_m1
CTGF	Hs00170014_m1
AREG	Hs00950669_m1
FN1	Hs01549976_m1
PDGFRA	Hs00998018_m1
PDGFA	Hs00234994_m1
PDGFRB	Hs01019589_m1
PDGFB	Hs00966522_m1
TGFBR1	Hs00610320_m1
TGFB1	Hs00998133_m1
COL1A1	Hs00164004_m1
ACTA2	Hs00426835_g1
MMP3	Hs00968305_m1
MMP9	Hs00957562_m1
TIMP1	Hs01092512_g1
TIMP2	Hs00234278_m1

Supplementary Table 3. Primers used for gene expression assays
