# Supplementary material for: Pulmonary fibrosis distal airway epithelia are dynamically and structurally dysfunctional
Source: Nat Commun. 2021 Jul 27;12:4566. doi: 10.1038/s41467-021-24853-8 (PMC8316442; doi:10.1038/s41467-021-24853-8)
Supplement: Supplementary file 3 — Description of Additional Supplementary Files [file 41467_2021_24853_MOESM3_ESM.docx]

**Description of Additional Supplementary Files**

**Supplementary Movie 1**

Control distal epithelia are unjammed at day 4 of ALI: unjammed control distal epithelia at day 4 of ALI, 200-minute-long video, images acquired every 5 minutes at 20x magnification, scale bar represents 100 $\mu$m.

**Supplementary Movie 2**

IPF distal epithelia are unjammed at day 4 of ALI: unjammed IPF distal epithelia at day 4 of ALI, 200-minute-long video, images acquired every 5 minutes at 20x magnification, scale bar represents 100 $\mu$m.

**Supplementary Movie 3**

Control distal epithelia are jammed at day 14 of ALI: jammed control distal epithelia at day 14 of ALI, 200-minute-long video, images acquired every 5 minutes at 20x magnification, scale bar represents 100 $\mu$m.

**Supplementary Movie 4**

IPF distal epithelia are unjammed at day 14 of ALI: unjammed IPF distal epithelia at day 14 of ALI, 200-minute-long video, images acquired every 5 minutes at 20x magnification, scale bar represents 100 $\mu$m.

**Supplementary Movie 5**

Untreated control distal epithelia persist in a jammed phase: jammed control distal epithelia filmed from days 14 – 16 of ALI, 48-hour-long video, images acquired every 20 minutes at 20x magnification, scale bar represents 100 $\mu$m.

**Supplementary Movie 6**

YAP-activation induces unjamming in control distal epithelia: XMU-MP-1 induced unjammed control distal epithelia filmed from days 14 – 16 of ALI, 48-hour-long video, images acquired every 20 minutes at 20x magnification, scale bar represents 100 $\mu$m.

**Supplementary Movie 7**

EGFR-activation induced unjamming in control distal epithelia: AREG induced unjammed control distal epithelia filmed from days 14 – 16 of ALI, 48-hour-long video, images acquired every 20 minutes at 20x magnification, scale bar represents 100 $\mu$m.

**Supplementary Movie 8**

Untreated IPF distal epithelia persist in an unjammed phase: unjammed IPF distal epithelia filmed from days 14 – 16 of ALI, 48-hour-long video, images acquired every 20 minutes at 20x magnification, scale bar represents 100 $\mu$m.

**Supplementary Movie 9**

YAP-inhibition induces jamming in IPF distal epithelia: Verteporfin induced jammed IPF distal epithelia filmed from days 14 – 16 of ALI, 48-hour-long video, images acquired every 20 minutes at 20x magnification, scale bar represents 100 $\mu$m.

**Supplementary Movie 10**

EGFR-inhibition induces jamming in IPF distal epithelia: AG-1478 induced jammed IPF distal epithelia filmed from days 14 – 16 of ALI, 48-hour-long video, images acquired every 20 minutes at 20x magnification, scale bar represents 100 $\mu$m.
