# Supplementary material for: Pulmonary fibrosis distal airway epithelia are dynamically and structurally dysfunctional
Source: Nat Commun. 2021 Jul 27;12:4566. doi: 10.1038/s41467-021-24853-8 (PMC8316442; doi:10.1038/s41467-021-24853-8)
Supplement: Supplementary file 14 — Reporting Summary [file 41467_2021_24853_MOESM14_ESM.pdf]

nature research

Corresponding author(s): David A. Schwartz

Last updated by author(s): Jun 3, 2021

Reporting Summary

Nature Research wishes to improve the reproducibility of the work that we publish. This form provides structure for consistency and transparency in reporting. For further information on Nature Research policies, see our Editorial Policies and the Editorial Policy Checklist.

Statistics

For all statistical analyses, confirm that the following items are present in the figure legend, table legend, main text, or Methods section.

n/a	Confirmed	
☐	☒	The exact sample size (*n*) for each experimental group/condition, given as a discrete number and unit of measurement
☐	☒	A statement on whether measurements were taken from distinct samples or whether the same sample was measured repeatedly
☐	☒	The statistical test(s) used AND whether they are one- or two-sided<br>*Only common tests should be described solely by name; describe more complex techniques in the Methods section.*
☐	☒	A description of all covariates tested
☐	☒	A description of any assumptions or corrections, such as tests of normality and adjustment for multiple comparisons
☐	☒	A full description of the statistical parameters including central tendency (e.g. means) or other basic estimates (e.g. regression coefficient) AND variation (e.g. standard deviation) or associated estimates of uncertainty (e.g. confidence intervals)
☐	☒	For null hypothesis testing, the test statistic (e.g. *F*, *t*, *r*) with confidence intervals, effect sizes, degrees of freedom and *P* value noted<br>*Give P values as exact values whenever suitable.*
☒	☐	For Bayesian analysis, information on the choice of priors and Markov chain Monte Carlo settings
☒	☐	For hierarchical and complex designs, identification of the appropriate level for tests and full reporting of outcomes
☒	☐	Estimates of effect sizes (e.g. Cohen's *d*, Pearson's *r*), indicating how they were calculated

Our web collection on statistics for biologists contains articles on many of the points above.

Software and code

Policy information about availability of computer code

Data collection	Time-lapse images were analyzed using PIVLab and custom scripts in MATLAB (R2019a). Cell shape data were collected using Seedwater segmenter (v0.5.7.1) in Python (v2.7) and ImageJ (v1.52). Gene analysis was completed with RStudio (v1.1.463). All other analysis was completed with GraphPad/PRISM (v8.3). RNA-Seq data has been deposited in the Gene Expression Omnibus. Accession No: GSE176001
Data analysis	Custom code in MATLAB was used to analyze epithelial dynamics. RNASeq data were analyzed using RStudio following established pipelines. Cell shape analysis occurred using Seedwater segmenter and ImageJ. All software available upon request.

For manuscripts utilizing custom algorithms or software that are central to the research but not yet described in published literature, software must be made available to editors and reviewers. We strongly encourage code deposition in a community repository (e.g. GitHub). See the Nature Research guidelines for submitting code & software for further information.

Data

Policy information about availability of data

All manuscripts must include a data availability statement. This statement should provide the following information, where applicable:

- Accession codes, unique identifiers, or web links for publicly available datasets
- A list of figures that have associated raw data
- A description of any restrictions on data availability

All data is available in the main text or the supplementary materials. All findings from the study are available upon request to the corresponding author. Source data are provided with this paper.

Field-specific reporting

Please select the one below that is the best fit for your research. If you are not sure, read the appropriate sections before making your selection.

- [x] Life sciences
- [ ] Behavioural & social sciences
- [ ] Ecological, evolutionary & environmental sciences

For a reference copy of the document with all sections, see nature.com/documents/nr-reporting-summary-flat.pdf

Life sciences study design

All studies must disclose on these points even when the disclosure is negative.

Sample size	Sample size analysis was not performed, but followed previous studies selection of multiple donors (N>=3 for all experiments performed) as is standard with other primary airway epithelial cultures (PMID: 26237129, 33028821, and 32117962). Phenotypes were consistent across all donors tested therefore we decided an adequate sample size had been achieved.
Data exclusions	No data were excluded from our analysis.
Replication	Experiments were carried out on healthy or diseased (IPF or COPD) human airway epithelial cells. Data was replicated across all samples that were tested. The cumulative total of cells used for these studies were as follows: healthy N = 11, IPF = 7, and COPD = 4. Each individual cell donor was repeated at least 3 independent times per condition with at least N>=2 technical replicates per donor for each experimental outcome.
Randomization	Internal controls were utilized for all experiments and each donor. Wells were assigned treatment conditions randomly (i.e. control: untreated, amphiregulin, or XMU-MP-1) and a vehicle control was utilized for all experiments (i.e. DMSO).
Blinding	A de-identified code was generated for each donor and only after experimentation and analysis was this code matched with donor information when comparing across diseases (i.e. healthy vs IPF vs COPD). The same format was utilized for control cell analysis for MUC5B snp - snp status was only identified after experimentation. For immunofluorescence and gene expression blinding was not possible as the same investigator performed and analyzed the experiments.

Reporting for specific materials, systems and methods

We require information from authors about some types of materials, experimental systems and methods used in many studies. Here, indicate whether each material, system or method listed is relevant to your study. If you are not sure if a list item applies to your research, read the appropriate section before selecting a response.

Materials & experimental systems

n/a	Involved in the study	
[ ]	[x]	Antibodies
[ ]	[x]	Eukaryotic cell lines
[x]	[ ]	Palaeontology and archaeology
[x]	[ ]	Animals and other organisms
[ ]	[x]	Human research participants
[x]	[ ]	Clinical data
[x]	[ ]	Dual use research of concern

Methods

n/a	Involved in the study	
[x]	[ ]	ChIP-seq
[x]	[ ]	Flow cytometry
[x]	[ ]	MRI-based neuroimaging

Antibodies

Antibodies used	The follow antibodies were used for immunofluorescence:<br>rabbit monoclonal anti-ERBB2 (Cell Signaling, 2165, 1:250), chicken polyclonal anti-KRT5 (BioLegend, 905901, 1:500), mouse monoclonal anti-Ki67 (Cell Signaling, 9449, 1:500), mouse monoclonal anti-MUC5B (Novus Biologics, NBP2-50390, 1:5000), rabbit monoclonal anti-Vimentin (Cell Signaling, 5741, 1:100), rabbit monoclonal anti-YAP (Cell Signaling, 14074, 1:250), mouse monoclonal anti-FOXJ1 (Invitrogen, 14-9965-82, 1:500), goat polyclonal anti-FOXJ1 (R&D Systems, AF3619, 1:500), mouse monoclonal anti-MUC5AC (Invitrogen, MA5-12178, 1:200), rat monoclonal anti-SCGB1A1 (R&D, MAB4218, 1:500), rabbit monoclonal anti-KRT8 (Abcam, ab53280, 1:100), rabbit monoclonal P-63\alpha (Cell Signaling 13109, 1:500), mouse monoclonal anti-alpha smooth muscle actin (Abcam, ab7817, 1:500), Phalloidin-iFluor (Abcam, ab176753, 1:2000).<br><br>All secondary antibodies were purchased from ThermoFisher and used at a concentration of 1:500:<br>Streptavidin 488 conjugate (Invitrogen, S11223), Donkey anti-rabbit IgG 488 (Invitrogen, A32790), Goat anti-rabbit IgG 488 (Invitrogen, A32731), Donkey anti-mouse IgG 488 (Invitrogen, A32766), Goat anti-mouse IgG 488 (Invitrogen, A32723), Goat anti-rabbit IgG 555 (Invitrogen, A-21428), Goat anti-mouse IgG 555 (Invitrogen, A-21422), Goat anti-chicken IgY 555 (Invitrogen, A32932), Streptavidin, 647 conjugate (Invitrogen, S21374), Donkey anti-rabbit IgG 647 (Invitrogen, A32795), and Donkey anti-mouse IgG 647 (Invitrogen, A32787)

For western blotting, we used the following antibodies: rabbit monoclonal anti-E-cadherin (Cell Signaling, 3195, 1:1000), rabbit monoclonal anti-N-cadherin (Cell Signaling, 13116, 1:1000), rabbit monoclonal anti-Snail (Cell Signaling, 3879, 1:1000, and goat polyclonal anti-beta-actin (Abcam, ab8229, 1:500).

Western blot secondary antibodies were purchased from LICOR and used at a concentration of 1:10,000:
IRDye 800CW Donkey anti-Rabbit IgG (LICOR, 926-32213) and IRDye 800CW Donkey anti-Goat IgG (LICOR, 926-32214)

Validation

All antibodies were manufacturer validated and commercial available. Validation statements on manufacturer website listed below:

Immunofluorescence
anti-ERBB2 (https://www.cellsignal.com//products/primary-antibodies/her2-erbb2-29d8-rabbit-mab/2165)
anti-KRT5 (https://www.biolegend.com/en-us/products/purified-anti-keratin-5-polyclonal-chicken-antibody-15091)
anti-Ki67 (https://www.cellsignal.com//products/primary-antibodies/ki-67-8d5-mouse-mab/9449)
anti-MUC5B (https://www.novusbio.com/products/muc5b-antibody-6f10-e4_nbp2-50390)
anti-Vimentin (https://www.cellsignal.com//products/primary-antibodies/vimentin-d21h3-xp-rabbit-mab/5741)
anti-YAP (https://www.cellsignal.com//products/primary-antibodies/yap-d8h1x-xp-rabbit-mab/14074)
anti-FOXJ1 (https://www.thermofisher.com/antibody/product/FOXJ1-Antibody-clone-2A5-Monoclonal/14-9965-82)
anti-FOXJ1 (https://www.fishersci.com/shop/products/anti-human-foxj1-polyclonal-r-d-systems/AF3619)
anti-MUC5AC (https://www.thermofisher.com/antibody/product/MUC5AC-Antibody-clone-45M1-Monoclonal/MA5-12178)
anti-SCGB1A1 (https://www.rndsystems.com/products/human-uteroglobin-scgb1a1-antibody-394324_mab4218)
anti-KRT8 (https://www.abcam.com/cytokeratin-8-antibody-ep1628y-cytoskeleton-marker-ab53280.html)
anti-P63 (https://www.cellsignal.com//products/primary-antibodies/p63-a-d2k8x-xp-rabbit-mab/13109)
anti-alpha smooth muscle actin (https://www.abcam.com/alpha-smooth-muscle-actin-antibody-1a4-ab7817.html)

Immunofluorescence secondary:
Streptavidin 488 conjugate (https://www.thermofisher.com/order/catalog/product/S11223#/S11223)
Donkey anti-rabbit IgG 488 (https://www.thermofisher.com/antibody/product/Donkey-anti-Rabbit-IgG-H-L-Highly-Cross-Adsorbed-Secondary-Antibody-Polyclonal/A32790)
Goat anti-rabbit IgG 488 (https://www.thermofisher.com/antibody/product/Goat-anti-Rabbit-IgG-H-L-Highly-Cross-Adsorbed-Secondary-Antibody-Polyclonal/A32731)
Donkey anti-mouse IgG 488 (https://www.thermofisher.com/antibody/product/Donkey-anti-Mouse-IgG-H-L-Highly-Cross-Adsorbed-Secondary-Antibody-Polyclonal/A32766)
Goat anti-mouse IgG 488 (https://www.thermofisher.com/antibody/product/Goat-anti-Mouse-IgG-H-L-Highly-Cross-Adsorbed-Secondary-Antibody-Polyclonal/A32723)
Goat anti-rabbit IgG 555 (https://www.thermofisher.com/antibody/product/Goat-anti-Rabbit-IgG-H-L-Cross-Adsorbed-Secondary-Antibody-Polyclonal/A-21428)
Goat anti-mouse IgG 555 (https://www.thermofisher.com/antibody/product/Goat-anti-Mouse-IgG-H-L-Cross-Adsorbed-Secondary-Antibody-Polyclonal/A-21422)
Goat anti-chicken IgY 555 (https://www.thermofisher.com/antibody/product/Goat-anti-Chicken-IgY-H-L-Cross-Adsorbed-Secondary-Antibody-Polyclonal/A32932)
Streptavidin, 647 conjugate (https://www.thermofisher.com/order/catalog/product/S21374#/S21374)
Donkey anti-rabbit IgG 647 (https://www.thermofisher.com/antibody/product/Donkey-anti-Rabbit-IgG-H-L-Highly-Cross-Adsorbed-Secondary-Antibody-Polyclonal/A32795)
Donkey anti-mouse IgG 647 (https://www.thermofisher.com/antibody/product/Donkey-anti-Mouse-IgG-H-L-Highly-Cross-Adsorbed-Secondary-Antibody-Polyclonal/A32787)

Western blot:
anti-E-cadherin (https://www.cellsignal.com//products/primary-antibodies/e-cadherin-24e10-rabbit-mab/3195)
anti-N-cadherin (https://www.cellsignal.com//products/primary-antibodies/n-cadherin-d4r1h-xp-rabbit-mab/13116)
anti-Snail (https://www.cellsignal.com//products/primary-antibodies/snail-c15d3-rabbit-mab/3879)
anti-beta-actin (https://www.abcam.com/beta-actin-antibody-loading-control-ab8229.html)

Western blot secondary:
IRDye 800CW Donkey anti-Mouse IgG (https://www.licor.com/bio/reagents/irdye-800cw-donkey-anti-mouse-igg-secondary-antibody)
IRDye 800CW Donkey anti-Rabbit IgG (https://www.licor.com/bio/reagents/irdye-800cw-donkey-anti-rabbit-igg-secondary-antibody)
IRDye 800CW Donkey anti-Goat IgG (https://www.licor.com/bio/reagents/irdye-800cw-donkey-anti-goat-igg-secondary-antibody)

Eukaryotic cell lines

Policy information about cell lines

Cell line source(s)

We used primary human distal and bronchial airway epithelial cells and lung fibroblasts derived from patient explants.

Authentication

The cells were not authenicated

Mycoplasma contamination	The cells were not tested for mycoplasma
Commonly misidentified lines (See ICLAC register)	There were no commonly misidentified cell lines utilized in this study.

Human research participants

Policy information about studies involving human research participants

Population characteristics	Primary human epithelial cells and fibroblasts were obtained from two sources (1) provided by Dr. Hong Wei Chu at National Jewish Health, or (2) lung transplant collection at the University of Colorado Hospital (IRB protocol: 11-1664 or 18-0572). Control cells were collected from lungs not suitable for transplantation or from IPF or COPD explanted lungs. Clinical diagnosis was established for IPF or COPD patients. Informed consent was obtained from all patients. Demographic information is provided in the manuscript. All patient samples were control for age and smoking history.
Recruitment	Potential participants were identified in the Adult Pulmonary Clinics (Center for Lungs and Breathing at UCH), Thoracic Surgery Clinic, Transplant Clinic, and Inpatient Service. All subjects who meet the inclusion criteria were eligible for study participation. The UCH medical record was pre-screened before clinic to facilitate recruitment and enrollment. There was no self-selection bias in this study.
Ethics oversight	Tissue samples were obtained under protocol 11-1664 and 18-0572 approved by University of Colorado Anschutz Medical campus institutional review board.

Note that full information on the approval of the study protocol must also be provided in the manuscript.
